# Nanoparticulate Transport of Oximes over an *In Vitro* Blood-Brain Barrier Model

**DOI:** 10.1371/journal.pone.0014213

**Published:** 2010-12-03

**Authors:** Sylvia Wagner, Jürgen Kufleitner, Anja Zensi, Miriam Dadparvar, Sascha Wien, Judith Bungert, Tikva Vogel, Franz Worek, Jörg Kreuter, Hagen von Briesen

**Affiliations:** 1 Department of Cell Biology and Applied Virology, Fraunhofer Institute for Biomedical Engineering, Sankt Ingbert, Germany; 2 Institute of Pharmaceutical Technology, Goethe-University, Frankfurt am Main, Germany; 3 Biotechnology General, Rehovot, Israel; 4 Bundeswehr Institute of Pharmacology und Toxicology, München, Germany; The University of Akron, United States of America

## Abstract

**Background:**

Due to the use of organophosphates (OP) as pesticides and the availability of OP-type nerve agents, an effective medical treatment for OP poisonings is still a challenging problem. The acute toxicity of an OP poisoning is mainly due to the inhibition of acetylcholinesterase (AChE) in the peripheral and central nervous systems (CNS). This results in an increase in the synaptic concentration of the neurotransmitter acetylcholine, overstimulation of cholinergic receptors and disorder of numerous body functions up to death. The standard treatment of OP poisoning includes a combination of a muscarinic antagonist and an AChE reactivator (oxime). However, these oximes can not cross the blood-brain barrier (BBB) sufficiently. Therefore, new strategies are needed to transport oximes over the BBB.

**Methodology/Principal Findings:**

In this study, we combined different oximes (obidoxime dichloride and two different HI 6 salts, HI 6 dichloride monohydrate and HI 6 dimethanesulfonate) with human serum albumin nanoparticles and could show an oxime transport over an *in vitro* BBB model. In general, the nanoparticulate transported oximes achieved a better reactivation of OP-inhibited AChE than free oximes.

**Conclusions/Significance:**

With these nanoparticles, for the first time, a tool exists that could enable a transport of oximes over the BBB. This is very important for survival after severe OP intoxication. Therefore, these nanoparticulate formulations are promising formulations for the treatment of the peripheral and the CNS after OP poisoning.

## Introduction

The extensive use of organophosphates (OP) as insecticides and pesticides in agriculture and for suicide attempt causes worldwide several hundreds of thousands intoxications and fatalities per year [1], [2], [3], [4], [5]. Hereby, mild poisonings are mainly caused by accidental exposure [4], [6], [7], [8] and severe cases are mostly due to suicidal use [9], [10]. In addition, highly toxic OP such as tabun, sarin, soman and VX, also known as ‘nerve agents’, are the deadliest chemical weapons so far. The repetitive use of nerve agents during military conflicts and by terrorists [11] emphasizes a lingering threat to the population. Therefore, the necessity still exists to develop an effective medical treatment for OP poisonings. The acute toxic effects of OP is mainly due to a progressive inhibition of acetylcholinesterase (AChE) by phosphylation (phosphorylation and phosphonylation) of their active serine center leading to an inactive enzyme species [12], [13], [14] in the peripheral as well as the central nervous systems (CNS) [15]. AChE normally regulates the concentration of the neurotransmitter acetylcholine (ACh) [16]. Phosphylation of the active serine site leads to an accumulation of the synaptic ACh concentration, followed by overstimulation of cholinergic receptors and disorder of numerous body functions such as salivation, lacrimation, tremors, miosis, diarrhea, and, at worst, respiratory difficulties and death due to anoxia. OP induced brain injury is characterized by rapid loss of consciousness, seizures, inhibition of the respiratory centers in the medulla oblongata as well as long-term behavioral changes in sub-lethal injuries [15]. The pharmacological standard treatment of OP poisoning includes a combination of a muscarinic antagonist, e.g. atropine, and an AChE reactivator (oxime), e.g. pyridine 2-aldoxime (2-PAM) or obidoxime, to repair the biochemical lesion by dephosphylating the enzyme and returning the function of AChE [15]. However, due to the chemical nature of the oximes and their pharmacokinetic profile these oximes can hardly cross the blood-brain barrier (BBB) and do not reach the central compartment in a sufficient manner [17], [18], [19], [20], [21]. Thus, oximes may reactivate phosphylated AChE at peripheral sites but marginal in the brain. Therefore, new strategies are needed to transport oximes over the BBB.

The BBB is created by the cerebral endothelial cells which function as the major exchange interface between the blood and the brain. Tight Junctions (TJ) close the intracellular space between the endothelial cells and block the free diffusion of water-soluble polar substances. The BBB protects the brain from the peripheral circulation and toxic substances and restricts the transport of many therapeutically important drugs from the blood into the brain. Therefore, the BBB represents an insurmountable obstacle for the effective treatment of many brain diseases.

Over the past few years a number of different strategies have been devised to overcome this barrier such as osmotic opening of the TJ and the direct surgical administration of drugs into the brain. However, the most notable progress has been achieved by the use of nanotechnology. Different polymeric nanoparticles [22] as well as solid lipid nanoparticles and liposomes have successfully been used for the transport of drugs across the BBB and into the brain. It was possible to transport more than 10 different nanoparticle-bound drugs including doxorubicin, dalargin, loperamide, tubocurarin, nerve growth factor with different chemical properties and pharmacological effects over the BBB. Furthermore, these nanocarriers have not only enhanced the transport of the drug into the brain, they also protected the active agent from enzymatic degradation, and they were able to reduce side effects [23]. The mechanism of the nanoparticulate drug transport over the BBB has not been fully understood so far and has been discussed controversially. It has been suggested that nanoparticle systems exert a generalized toxic effect on the BBB by opening the TJ and allowing paracellular movement of solutes into the brain [24]. However, later studies have clearly shown that no toxic effects occurred, *in vivo* and *in vitro*, at normal dosages. These studies clearly demonstrated that the nanoparticles enable a transport of drugs over the BBB and that an association of the drugs with the nanoparticles is a requirement for this transport [25]. Furthermore, it has been hypothesized that after injection into the blood stream apolipoproteins are adsorbed onto the surface of polysorbate 80-coated nanoparticles [26]. Thus, a number of transport mechanisms for lipoproteins over the BBB exist [27], [28]. The apolipoprotein-modified particles could then interact with apolipoprotein receptors at the BBB and result in their endocytotic uptake into endothelium and possibly transcytosis into the brain. This suggestion is supported by the observation that nanoparticles made of human serum albumin (HSA) with adsorbed or covalently bound apolipoprotein E [29] or apolipoprotein AI [30] transported attached drugs equally well across the BBB [31]. However, running mechanism studies showed a specific binding and uptake of apolipoprotein-modified HSA nanoparticles on endothelial cells. Moreover, they are able to enter the CNS by transcytosis and are delivered to neurons [29].

In the present study, obidoxime dichloride and both HI 6 salts HI 6 dichloride monohydrate and HI 6 dimethanesulfonate were bound to biodegradable HSA nanoparticles and the oxime transport over an *in vitro* BBB model as well as the reactivation of OP-inhibited AChE in this model were assessed. The nanoparticulate transported oximes achieved an even better reactivation of OP-inhibited AChE than freely diffusing oximes.

## Materials and Methods

### Nanoparticle preparation and characterization

#### Preparation of HSA nanoparticles

HSA nanoparticles were prepared by a desolvation technique previously described by Langer et al. [32]. Briefly, 200 mg HSA (fraction V; purity 96–99%) were dissolved in 2.0 ml 10 mM sodium chloride. After pH adjustment to pH 8.3, the desolvation was performed by drop-wise addition of 8.0 ml 96% Ethanol under stirring (600 rpm) at room temperature (RT). To stabilize the particles by crosslinking, 117.5 µl 8% glutaraldehyde were added. After 18 h of constant stirring, the resulting nanoparticle suspension was purified by centrifugation (16,100×g, 8 min) and redispersion of the pellet in water.

Recombinant apolipoprotein E (ApoE) (34,200 Da, Biotechnology General, Rehovot, Israel) was attached covalently to the nanoparticles via a bifunctional NHS-PEG-Mal crosslinker (5,000 Da, RAPP Polymere GmbH, Tübingen, Germany) which reacts with amino groups on the surface of the particles as well as with thiol groups introduced into the ApoE molecule [33]. Therefore, the nanoparticles were centrifuged and redispersed in phosphate buffer pH 8.0. A ten-fold molar excess of the NHS-PEG-Mal linker was added to the nanoparticles for the activation reaction under constant shaking for 1 h at RT. Then, these activated nanoparticles were purified by centrifugation and redispersion as described above.

In order to introduce sulfhydryl groups into the apolipoprotein molecule, ApoE was dissolved in phosphate buffer pH 8.0, and a 50-fold molar excess of 2-iminothiolane was added. This mixture was incubated for 2 h under constant shaking at RT. The purification of thiolated apolipoprotein was performed by size exclusion chromatography with a D-Salt™ desalting column (Pierce, Rockford, USA) as previously described by Michaelis et al. [33]. For the conjugation of the activated nanoparticles with the thiolated ApoE, 1 ml of thiolated apolipoprotein was added to 1 ml of activated nanoparticles and the mixture was stirred (650 rpm) for 12 h at RT. These conjugated nanoparticles were purified as described above. Supernatants were collected to determine the amount of unbound ApoE by a standard Micro BCA™ protein assay (Pierce, Rockford, USA) according to the manufacturer's instructions.

As a control, unmodified HSA nanoparticles were associated with poly-ethylene-glycol (PEG) chains by linking mPEG-SPA-5000 (Nektar, Huntsville, USA) to the particle surface. The nanoparticles were incubated with a 50-fold molar excess of mPEG-SPA-5000 in phosphate buffer pH 8.0 for 1 h under constant shaking at RT. These PEGylated nanoparticles were purified as described above.

#### Drug-loading of the nanoparticles

The nanoparticles were loaded with the drug by adsorption on particles surface. The adsorptive drug-loading of the nanoparticles was freshly performed 1 day before use by adding of the different oxime solutions (obidoxime dichloride, HI 6 dichloride monohydrate and HI 6 dimethanesulfonate, respectively) to the nanoparticles. The adsorption occurred under stirring (650 rpm) at RT over night and the adsorptive drug-loaded nanoparticles were used directly for the experiments.

### Nanoparticle characterization

The concentration of HSA nanoparticles was determined by microgravimetry [34].

The mean particle diameter and polydispersity were investigated by photon correlation spectroscopy (PCS) using a Malvern Zetasizer 3000 HS_A_ (Malvern Instruments Ltd., Malvern, UK).

### Isolation of primary porcine brain capillary endothelial cells (pBCEC) and cell culture

#### Isolation of primary porcine brain capillary endothelial cells (pBCEC)

Primary porcine brain capillary endothelial cells (pBCEC) were isolated according to a modified version of the method of Zenker et al. [35]. Briefly, fresh porcine skulls were obtained from the local slaughterhouse (Schlachthof Zweibrücken, Germany) after routine slaughter. Animals were killed in accordance to the guideline 93/119/EC of the European Community on the protection of animals at the time of slaughter or killing, dated December 22nd, 1993. After opening of the skullcap cerebral tissues were prepared. The meninges were removed and the grey matter was collected and minced into small pieces. pBCEC were isolated by two enzymatic digestion and centrifugation steps with dispase and collagenase and a further digestion step with DNase. Afterwards, large macro-vascular blood vessels and incomplete digested capillary fragments were removed by filtration through a cell strainer and resting erythrocytes were lysed.

pBCEC were cultivated on collagen IV-coated flasks, plates or Transwell systems at 37°C and 5% CO_2_ in M199 medium, supplemented with 10% newborn calf serum, 0.7 mM L-glutamine and antibiotics (penicillin/streptomycin and gentamicin). The first medium change to remove debris from attached cells occurred 1 h and 24 h after plating, if the cells grew on Transwells or 24 h after plating, if the cells grew on cell culture flasks or plates. The medium was switched to serum-free medium (DMEM/F12 with L-glutamine, penicillin/streptomycin, gentamicin and 550 nM hydrocortisone) after the cells reached confluence.

#### Cell culture

Furthermore, the mouse brain endothelioma cell line bEnd3 (LGC Promochem, Wesel, Germany) was used. The cells were cultured at 37°C and 5% CO_2_ in DMEM high glucose medium, supplemented with 10% fetal calf serum.

### Cellular uptake and intracellular distribution of the nanoparticles

Cellular uptake and intracellular distribution of the nanoparticles were studied by confocal laser scanning microscopy (CLSM). bEnd3 cells were cultured on collagen IV-coated glass slides and treated with the different nanoparticle formulations for 4 h at 37°C. After this incubation the cells were washed twice with serum-free medium and the cytosol was stained with CellTracker™ Red CMTPX (Invitrogen, Karlsruhe, Germany) as described in the manufacturer instructions manual. Cells were fixed with 0.5–1% PFA for 5–10 min. After fixation the cells were embedded in Vectashield HardSet Mounting Medium containing DAPI for cell nuclei staining. The CLSM study was performed with an Axiovert 200 M microscope with a 510 NLO Meta device (Zeiss, Jena, Germany), Ti:Sa femtosecond or an argon ion laser and the LSM Image Examiner software. The green autofluorescence of the nanoparticles at 488/520 nm was used here.

### Cell viability study

Cell viability was determined in pBCEC using WST-1 assay (Cell Proliferation Reagent, Roche Diagnostics, Mannheim, Germany) based upon the absorption measurement of formazan formation. Briefly, cells were cultured in 96-well plates under the same conditions as for drug transport study and were incubated with the different nanoparticulate formulations for 24 h at 37°C. Afterwards, the cells were washed and the determination of cell viability was carried out after addition of WST-1 reagent and formazan measurement, as described in the manufacturer instructions manual.

### Measurement of transendothelial electrical resistance (TER) and the capacitance (C_cl_)

After the isolation of the pBCEC, the cells were seeded on collagen IV-coated Transwell inserts, which were placed in the cellZscope (nanoAnalytics, Münster, Germany). The transendothelial electrical resistance (TER) and the capacitance (C_cl_) of the barrier-forming pBCEC were measured automatically every hour under physiological conditions by impedance measurement with the cellZscope, which means that the cell module of the cellZscope is placed inside of the incubator during the experiment and the external controller is connected to a computer outside of the incubator. Therefore, there is no influence of temperature changes or vibration during the TER measurement.

### Drug transport study

#### Preparation of human AChE

Haemoglobin-free erythrocyte ghosts, as a source of membrane-bound human AChE, were prepared according to Dodge et al. [36] with minor modifications according to [37]. Briefly, heparinized human blood was centrifuged, the plasma removed and the erythrocytes were washed with phosphate buffer (0.1 M, pH 7.4). The packed erythrocytes were diluted in hypotonic phosphate buffer (6.7 mM, pH 7.4) to facilitate haemolysis, followed by centrifugation. The supernatant was removed and the pellet resuspended in phosphate buffer (0.1 M, pH 7.4), after additional washing cycles. Aliquots of the erythrocyte ghosts were stored at −80°C until use.

#### Inhibition of AChE

OP-inhibited AChE was prepared by incubation of AChE with paraoxon-ethyl or sarin for 15 min at 37°C. The OP excess after inhibition was removed by overnight dialyses with phosphate buffer (0.1 M, pH 7.4) at 4°C [37]. The OP-inhibited AChE was stored at −80°C until use.

#### Enzymatic assay

For drug transport studies the pBCEC were seeded on collagen IV-coated Transwell inserts directly after isolation. The cells were incubated with the different nanoparticulate formulations for 4 h at 37°C by adding the nanoparticles into the upper/apical compartment of the Transwell system, when the TER was at the maximum. After 4 h the medium of the lower/basolateral compartment was collected and the transport of the drug was measured by the reactivation of OP-inhibited AChE as earlier described in [37]. Briefly, at 0 min 10 µl aliquots of the inhibited AChE were transferred to cuvettes containing phosphate buffer (0.1 M, pH 7.4), 5,5′-dithio-bis-2-nitrobenzoic acid (DTNB) and 750 µl of the collected medium, which contains the transported oxime. After incubating for 15, 30, 45 and 60 min (at 37°C) the AChE activity was determined spectrophotometrically according to Ellman et al. [38] by adding acetylthiocholine iodide (ASCh). The assay was run at 37°C and 412 nm.

#### Reactivation kinetics

The reactivation of OP-inhibited AChE proceeds according to the following scheme 1 [37], [39]:



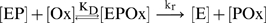
(Scheme 1)


In this scheme [EP] is the phosphylated AChE, [EPOx] the phosphyl-AChE-oxime complex, [Ox] the reactivator ( =  oxime), [E] the reactivated enzyme and [POx] the phosphylated oxime. K_D_ ( =  [EP] [Ox]/[EPOx]) is the dissociation constant, which is inversely proportional to the affinity of the oxime to [EP], and k_r_ the rate constant for the displacement of the phosphyl-residue from [EPOx] by the oxime, indicating the reactivity.

In case of a complete reactivation, with [Ox] » [EP]_0_, a pseudo-first-order rate equation 1 can be derived for reactivation process [40].
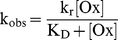
(1)


Using a discontinuous method for determination of enzyme activity after different reactivation times and setting the maximal reactivation of the enzyme as 100%, k_obs_ values can be calculated by non-linear regression analysis, applying equation 2:
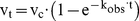
(2)


This equation represents the maximal velocity (v_c_) and the velocity at time t (v_t_).

#### Calculation of the oxime concentration

For calculation of the transported oxime v_c_, v_i_ (velocity of the inhibited enzyme) and v_t_ were measured with the enzymatic AChE-assay and k_obs_ was calculated according to equation 2. After rearrangement of equation 1, oxime concentration can be calculated according to equation 3:
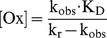
(3)


In the experimental set-up for this enzymatic AChE-assay K_D_ = 32.20 and k_r_ = 0.81, if obidoxime dichloride is used as reactivator together with paraoxon-ethyl as inhibitor and K_D_ = 50.10 and k_r_ = 0.677, if the HI 6 salts are used as reactivator together with sarin as inhibitor. The constants were taken from Worek et al. [37].

For the standardized oxime concentrations the dilution of the oxime in the cuvette and the Transwell system were taken into account.

The enhanced oxime-transport using the nanoparticulate formulations was calculated related to the transported free oximes and was expressed as transport difference (%).

Statistical analysis was made using the two-tailed Mann-Whitney U Test, which is equivalent to the Wilcoxon rank sum test [41], [42], [43]. Statistical significance was defined at P<0.05 values.

## Results and Discussion

### Nanoparticle preparation and characterization

The human serum albumin-based nanoparticles were prepared by a well known desolvation technique previously described by Langer et al. [32]. The size of the ApoE-modified nanoparticles was 229.9±15.8 nm and the size of the PEGylated nanoparticles was 217.6±14.0 nm. The polydispersity index of all preparations was smaller than 0.1 demonstrating that monodisperse nanoparticle formulations have been prepared. As shown by Micro BCA™ protein assay, 34.0±5.2% of the initial apolipoprotein amount was associated with the nanoparticles.

In order to minimize the very time-consuming and cost-intensive isolation of primary brain capillary endothelial cells (pBCEC) brain capillary endothelium cell lines, as the bEnd3, were used for cellular uptake and subcellular distribution studies. These cell lines still express the appropriate receptors of the BBB, but they already lost some other characteristics of the BBB, like a high transendothelial electrical resistance as indications of intact tight junctions [44], [45]. Also primary cells longer cultivated in cell culture lose their BBB characteristics [46] with the time. Therefore, cell viability and drug transport studies were done on freshly isolated primary cells accomplished after measurement of their transendothelial electrical resistance.

### Cellular uptake and subcellular distribution of the nanoparticles

**Figure 1:**
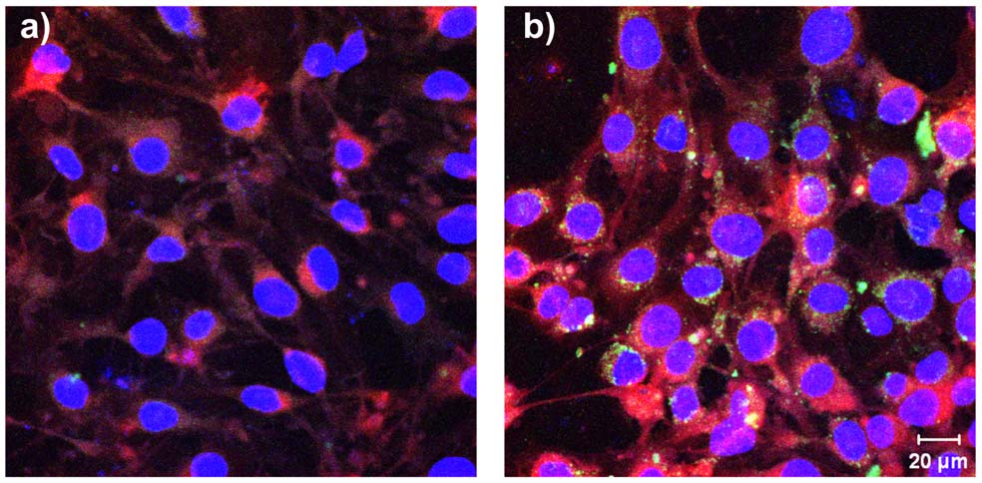
Cellular uptake and intracellular distribution of the nanoparticles studied by CLSM. bEnd3 cells were cultured on collagen IV-coated glass slides and were treated with a) PEGylated HI 6 dichloride monohydrate-loaded nanoparticles or b) ApoE-modified HI 6 dichloride monohydrate-loaded nanoparticles for 4 h at 37°C. The green autofluorescence of the nanoparticles was used for detection. Red: cytosol stained with CellTracker™ Red CMTPX, blue: nucleus stained with DAPI. Pictures were taken within inner sections of the cells.

The specificity of the cellular binding and uptake of the unloaded ApoE-modified nanoparticles in comparison to the PEGylated nanoparticles and the optimal incubation time of 4 h could be evidenced in our former study by Zensi et al. [29]. Therefore, the cellular uptake and intracellular distribution of the oxime-loaded ApoE-modified or PEGylated nanoparticulate formulations were exemplarily shown on HI 6 dichloride monohydrate-loaded nanoparticles by CLSM. bEnd3 cells were incubated either with 0.26 mg nanoparticles per cm^2^ growth area of the specific ApoE-modified or the corresponding PEGylated control nanoparticles for 4 h at 37°C (Figure 1). Pictures were taken within inner sections of the cells. Green fluorescence of specifically uptaken ApoE-modified nanoparticles was clearly visible within the inner part of the red stained cytosol (1b) whereas only some unspecific PEGylated nanoparticles can be detected (1a). Hence, the previous binding properties of the nanoparticles detected by FACS could be confirmed.

In summary, the shown CLSM data in combination with the previous FACS data clarifies the specific binding and intracellular uptake of the oxime-loaded ApoE-modified nanoparticulate formulations.

### Cell viability study

**Figure 2:**
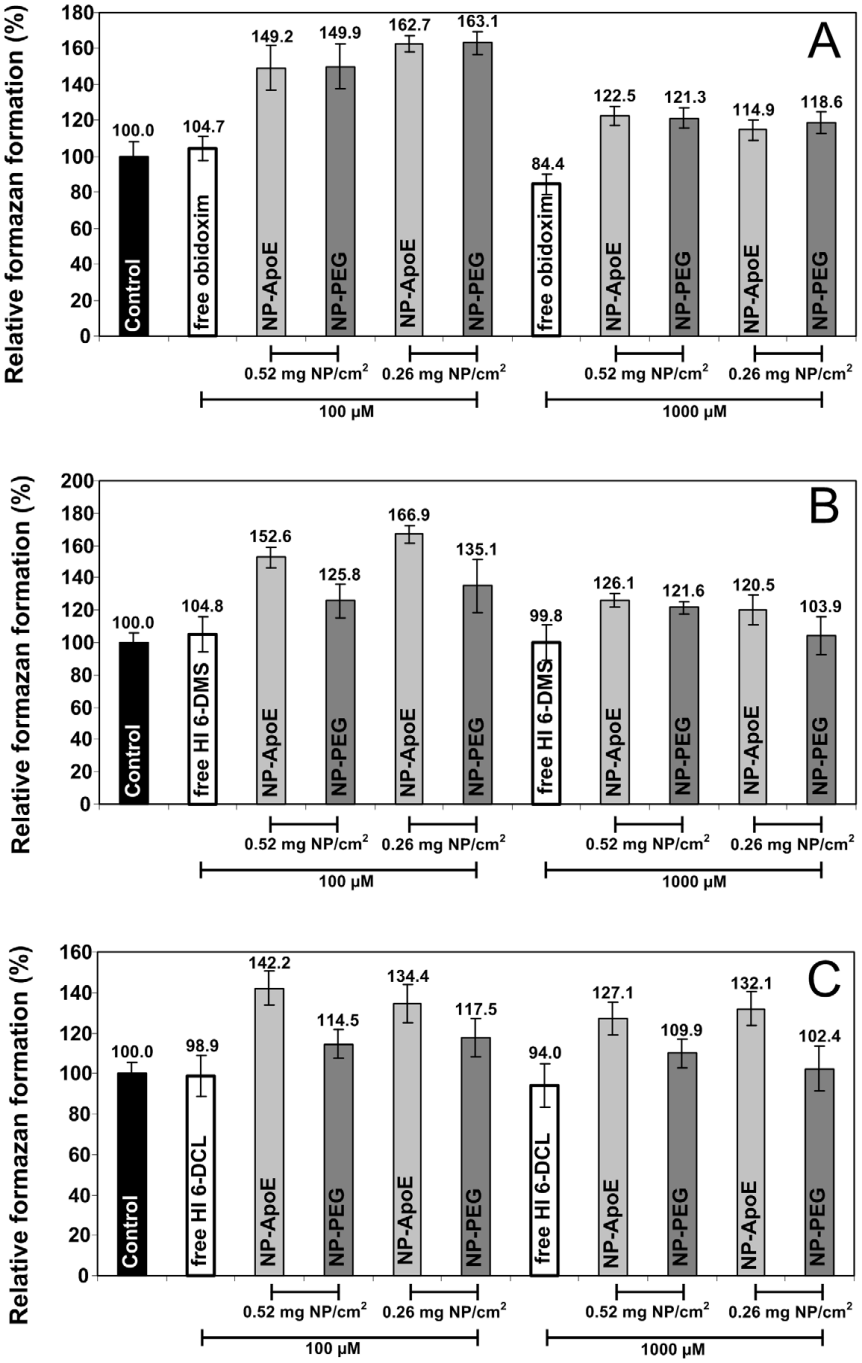
Cell viability assay. pBCEC were cultured in collagen IV-coated 96-well plates under the same conditions as for drug transport study. The cells were incubated for 24 h at 37°C with 0.26 mg nanoparticles per cm^2^ growth area (0.26 mg NP/cm^2^) or 0.52 mg nanoparticles per cm^2^ growth area (0.52 mg NP/cm^2^) of the different ApoE-modified (NP-ApoE) as well as the PEGylated (NP-PEG) nanoparticulate formulations, which were loaded with 100 µM or 1000 µM of obidoxime, HI 6 dichloride monohydrate (HI 6-DCL) or HI 6 dimethanesulfonate (HI 6-DMS). Afterwards, the viability was determined after addition of WST-1 reagent and measurement of formazan formation, as described in the manufacturer instructions manual. Untreated cells set the 100% standard.

To exclude any cytotoxic effects of the oxime-loaded nanoparticles and the free oximes on the endothelial cells, the cell viability was determined after incubation of the cells with the oxime-loaded nanoparticles and the free drug. All the different nanoparticle concentrations and oxime concentrations of each oxime (obidoxime dichloride and both HI 6 salts HI 6 dichloride monohydrate and HI 6 dimethanesulfonate) were tested. Therefore, pBCEC were cultured and incubated for 24 h at 37°C with the different nanoparticulate formulations under the same conditions and concentrations as for drug transport studies. Cell viability was determined using a WST-1 assay based on the absorbance measurement of formazan formation. The formazan formation is only possible in the mitochondria of healthy cells, therefore, untreated cells set the 100% standard. After the incubation with the oxime-loaded formulations the cells showed more than 100% formazan formation, meaning that the viability is equal to the control cells (Figure 2). Therefore, any cytotoxic effect of these nanoparticulate formulations could be excluded. The formazan formation of more than 100% compared to the untreated control cells probably is caused by an activation of the pBCEC after nanoparticle incubation, visible with both nanoparticlulate formulations, the ApoE-modified (NP-ApoE) as well as the PEGylated control-nanoparticles (NP-PEG). This could already be discovered by our former studies with the unloaded particle system [29]. In the case of incubation with the free oximes the cell viability is nearly 100% (between 84.4 and 104.8%), meaning the free oximes are not toxic for the cells in the used concentration range (Figure 2).

### Measurement of transendothelial electrical resistance (TER)

**Figure 3:**
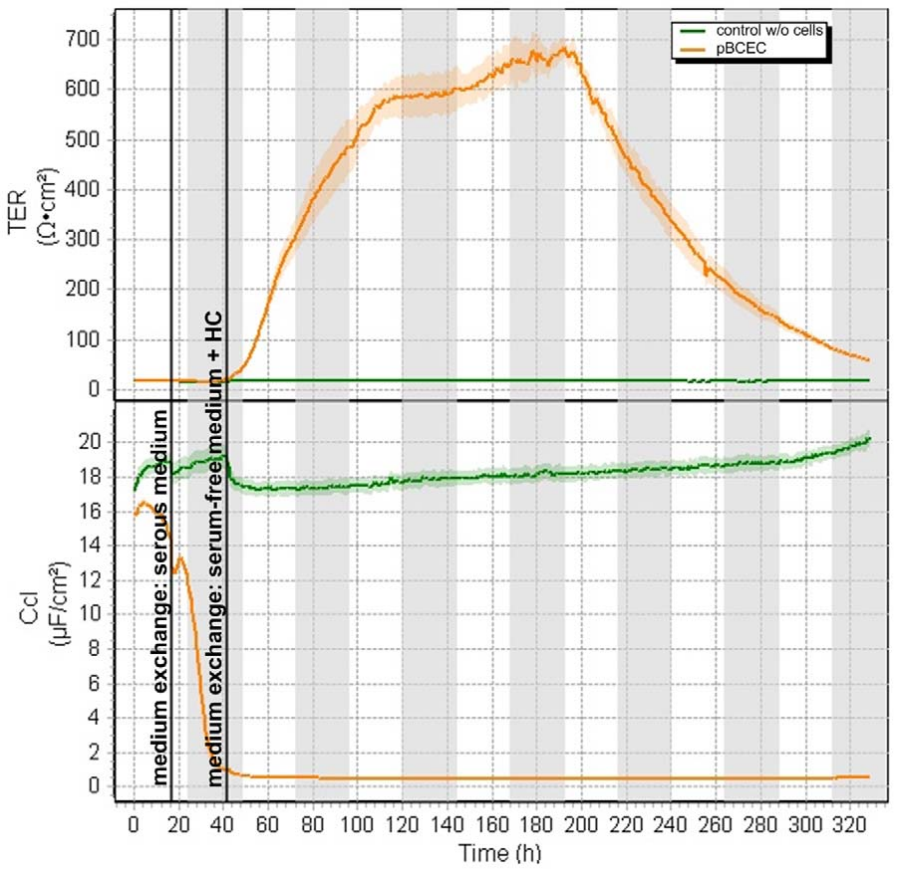
Measurement of transendothelial electrical resistance (TER) and the capacitance (C_cl_). pBCEC were seeded on collagen IV-coated Transwell inserts, which were placed in the cellZscope. The transendothelial electrical resistance (TER) and the capacitance (C_cl_) of the pBCEC were measured automatically every hour by impedance measurement. As control the measurement of the transendothelial electrical resistance (TER) of a Transwell insert without cells is shown.

Freshly isolated pBCEC are suitable for drug transport studies in the Transwell system, because micro-vascular endothelial cells develop also *in vitro* tight junctions (TJ), which constitute the integral component of the BBB characteristics. These TJ provide for a very limited and strongly adjusted material transfer between blood and brain and thus fulfill the barrier function. Due to the formation of the TJ *in vitro* a high transendothelial electrical resistance (TER) exists, which can be measured together with the capacitance (C_cl_) as indication for an adequate intact cell culture BBB model. The freshly isolated pBCEC were seeded on collagen IV-coated Transwell inserts and were cultivated with serous medium until C_cl_ decreased to 1 µF/cm^2^ and the TER started increasing, indicating a confluent monolayer. The medium was switched to serum-free hydrocortisone (HC)-containing medium after the cells reached confluence to force further increase of TER (Figure 3). When the TER reached its maximum and the values were at least over 300 Ωcm^2^ the nanoparticle incubation was started for drug transport studies.

**Figure 4:**
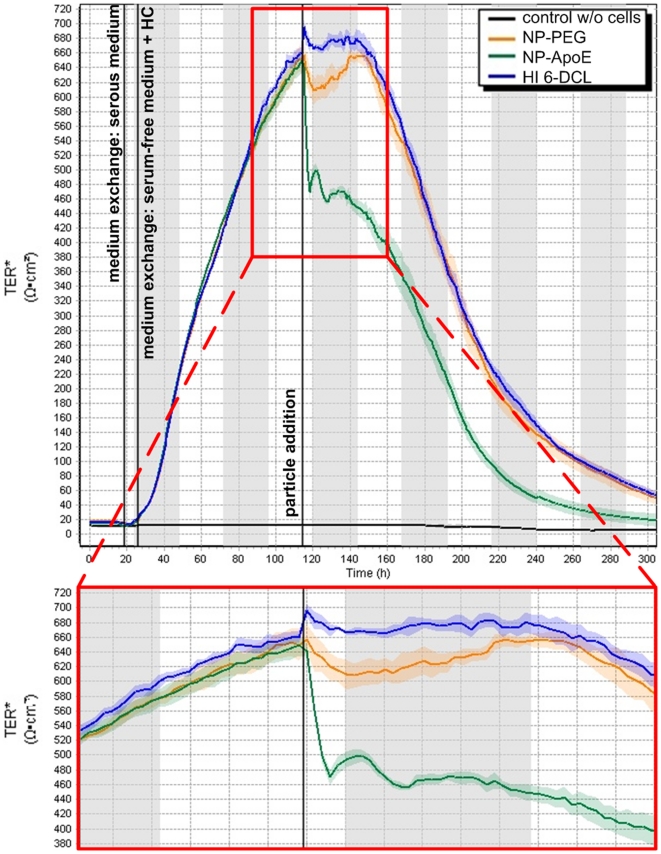
Long time measurement of transendothelial electrical resistance (TER) after nanoparticle addition. pBCEC were seeded on collagen IV-coated Transwell inserts and incubated with the free drug or 0.26 mg nanoparticles per cm^2^ growth area of the ApoE-modified (NP-ApoE) as well as the PEGylated (NP-PEG) nanoparticulate formulations, which were loaded with 1000 µM of HI 6 dichloride monohydrate (HI 6-DCL) at 37°C by adding the nanoparticles into the upper/apical compartment of the Transwell system. The TER was measured automatically every hour by impedance measurement. A magnification of the area of interest is highlighted in the red quadrangle. As control the measurement of the TER of a Transwell insert without cells is shown.

Furthermore, the measurement of the TER can be used for the valuation of the toxic potential of the nanoparticles on the BBB. Thus, 0.26 mg nanoparticles per cm^2^ growth area of the 1000 µM HI 6 dichloride monohydrate loaded specific ApoE-modified or the corresponding PEGylated nanoparticles as well as the free oxime were added ad the maximum of the TER values to the pBCEC to study the influence of the nanoparticles and the drug on the BBB properties (Figure 4). As already known, there is influence of the *in vitro* BBB properties by temperature changes or vibration like the nanoparticle incubation. Therefore, there was a minor transient break-in during the TER measurement after nanoparticle addition and a recovery of the TER could be observed (Figure 4). However, the TER remained on a high value, speaking for closed TJ and in consequence for a transcellular transportation route. Also, our former studies with the unloaded nanoparticles confirmed the integrity of the BBB after nanoparticle incubation in animal tests [29]. Therefore, this experiment supplemented the cell viability study and confirmed the non-toxicity of the nanoparticulate formulations.

### Drug transport study

For the drug transport studies the pBCEC were cultivated as described before. The cells were incubated with 0.26 mg or 0.52 mg nanoparticles per cm^2^ growth area (0.26 mg or 0.52 mg NP/cm^2^) of the specific ApoE-modified or the corresponding PEGylated control nanoparticles, which were adsorptively loaded with 100 µM or 1000 µM of the different oximes (obidoxime dichloride and the both HI 6 salts HI 6 dichloride monohydrate and HI 6 dimethanesulfonate). Furthermore, as control the cells were incubated with free oxime in the same amount as used for nanoparticle adsorption. After 4 h the medium of the lower/basolateral compartment was collected and the nanoparticulate drug transport was measured by the enzymatic AChE-assay as reactivation of the OP-inhibited AChE (see section 2.6.). The results, summarized in Tables 1–3, demonstrated that a nanoparticulate-mediated transport of active obidoxime, HI 6 dichloride monohydrate as well as HI 6 dimethanesulfonate over the *in vitro* BBB-model was possible with these nanoparticulate formulations. As expected, the specific ApoE-modified nanoparticles showed a better reactivation of the OP-inhibited AChE than the PEGylated control nanoparticles and free oximes, which can not cross the BBB in a sufficient manner. These findings argue for a nanoparticle-mediated transport and funneling of the adsorbed oxime over the BBB model. In all cases taking the standardized oxime concentration into account, the adsorptive loading with the higher oxime concentration (1000 µM compared to 100 µM) led to a better reactivation of the OP-inhibited AChE. Hereby, the nanoparticulate packaging with ApoE-modified nanoparticles was most effective for HI 6 dimethanesulfonate concerning the enhanced drug transport. Due to the individuality of primary cells after isolation the results of the drug transport experiments are normally not suitable to be averaged, as seen on the high standard deviation in Table 3, were it was exemplarily done once. Therefore, only the results of single experiments are shown on Tables 1–3. In the whole of the experiments the advantage of the ApoE-modified nanoparticles compared to the PEGylated particles and the free drug had to be recognized.

**Table 1:** Transport study of adsorptively obidoxime-loaded nanoparticles on an *in vitro* BBB model.

[NP], [drug-loading]	NP-ApoE		NP-PEG		free obidoxime
	standardized [oxime](µM)	transport difference(%)	standardized [oxime](µM)	transport difference(%)	standardized [oxime](µM)
0.52 mg NP/cm^2^, 100 µM	15.06	+39.06	13.01	+20.13	10.83
0.52 mg NP/cm^2^, 1000 µM	92.25	+133.49	63.47	+60.64	39.51
0.26 mg NP/cm^2^, 100 µM	9.06	−32.44	6.92	−48.40	13.41
0.26 mg NP/cm^2^, 1000 µM	39.22	+123.48	30.42	+73.33	17.55

**Table 2 pone-0014213-t002:** Transport study of adsorptively HI 6 dichloride monohydrate-loaded nanoparticles on an *in vitro* BBB model.

[NP], [drug-loading]	NP-ApoE		NP-PEG		free HI 6-DCL
	standardized [oxime](µM)	transport difference(%)	standardized [oxime](µM)	transport difference(%)	standardized [oxime](µM)
0.52 mg NP/cm^2^, 100 µM	25.76	+41.46	17.41	−4.39	18.21
0.52 mg NP/cm^2^, 1000 µM	61.81	+136.73	46.22	+77.02	26.11
0.26 mg NP/cm^2^, 100 µM	24.96	−3.89	5.13	80.25	25.97
0.26 mg NP/cm^2^, 1000 µM	67.97	+100.68	26.57	+26.57	33.87

**Table 3:** Transport study of adsorptively HI 6 dimethanesulfonate-loaded nanoparticles on an *in vitro* BBB model.

[NP], [drug-loading]	NP-ApoE		NP-PEG		free HI 6-DMS
	standardized [oxime](µM)	transport difference(%)	standardized [oxime](µM)	transport difference(%)	standardized [oxime](µM)
0.52 mg NP/cm^2^, 100 µM	25.91	+193.76	12.04	+36.51	8.82
0.52 mg NP/cm^2^, 1000 µM	44.59	+193.16	21.57	+41.81	15.21
0.26 mg NP/cm^2^, 100 µM	24.74	+143.50	8.76	−13.48	10.16
0.26 mg NP/cm^2^, 1000 µM	33.30±9.99 *	+123.26	23.50±6.33	−13.57	18.81±4.87 *

*Mann-Whitney U Test: significant difference between NP-ApoE and free HI 6-DMS (P<0.05).

These results demonstrated the cellular uptake of the specific ApoE-modified nanoparticles as well as the PEGylated control nanoparticles. In addition, a nanoparticle-mediated transport of different oximes over the *in vitro* BBB model consisting only of brain capillary endothelial cells was shown. However, a major difference appears to exist between ApoE-modified and PEGylated nanoparticles: Zensi et al. [29] showed that ApoE-modified nanoparticles were endocytosed and that they could cross the BBB *in vivo* and reached different brain regions, whereas PEGylated nanoparticles remained at the level of the endothelial cells and seemed not to penetrate the BBB. Even more importantly, Michaelis et al. [33] showed in *in vivo* experiments on mice, that only ApoE-modified nanoparticles were able to transport pharmacologically significant amounts of loperamide into the brain, not the PEGylated particles, whereas loperamide could not cross the BBB as free drug. Therefore, further *in vivo* experiments are needed to prove the transport efficiency of the nanoparticulate systems presented in this paper. Considering these findings it is most likely that only the ApoE-modified nanoparticles can transport a relevant amount of oximes specifically over the BBB.

The above studies demonstrated the proof of concept that a transport of oximes over a *in vitro* BBB model with adsorptively oxime-loaded nanoparticles was possible. Therefore, these particles could be suitable for the transport of oximes over the BBB, which is important for survival after OP intoxication. Hence, these nanoparticulate formulations are promising formulations for the treatment of the CNS and body after OP poisoning, which has to be tested *in vivo*. Furthermore, it has to be studied *in vivo* if the attachment of apolipoprotein E (ApoE) is necessary to enable the oximes to reach the acetylcholinesterase (AChE) sites in the brain.
